# Association between autonomic nervous dysfunction and cellular inflammation in end-stage renal disease

**DOI:** 10.1186/s12872-016-0385-1

**Published:** 2016-11-03

**Authors:** Eric Seibert, Kristina Zohles, Christof Ulrich, Alexander Kluttig, Sebastian Nuding, Jan A. Kors, Cees A. Swenne, Karl Werdan, Roman Fiedler, Matthias Girndt

**Affiliations:** 1 Department of Internal Medicine II, Martin Luther University Halle-Wittenberg, Halle (Saale), Germany; 2 Institute of Medical Epidemiology, Biostatistics, and Informatics, Martin Luther University Halle-Wittenberg, Halle (Saale), Germany; 3 Department of Internal Medicine III, Martin Luther University Halle-Wittenberg, Halle (Saale), Germany; 4 Department of Medical Informatics, Erasmus University Medical Center, Rotterdam, The Netherlands; 5 Department of Cardiology, Leiden University Medical Center, Leiden, The Netherlands

**Keywords:** Autonomic nerves, Dialysis, Inflammation, Heart rate variability, Monocytes

## Abstract

**Background:**

Alterations in autonomic nervous function are common in hemodialysis (HD) patients. Sympathetic as well as parasympathetic activation may be associated with immune and inflammatory responses. We intended to confirm a role of autonomous dysregulation for inflammation in HD patients.

**Methods:**

30 HD patients (including 15 diabetics) and 15 healthy controls were studied for heart rate variability (HRV) using 5 min ECG recordings. Heart rate variability was estimated by time-domain parameters (the standard deviation of the RR intervals (SDNN) and the percentage of pairs of adjacent RR intervals differing by >50 ms (pNN50)) and frequency-domain-analysis (high- and low-frequency variation of RR intervals, HF and LF). Inflammation was detected as serum C-reactive Protein (CRP), IL-6 and circulating monocyte subpopulation numbers. Immune cells were characterized by ACh receptor expression.

**Results:**

Patients differed from controls in terms of age (68.0 [14.8] yrs vs. 58.0 [13.0] yrs, *p* < 0.001; Median [IQR]) and sex. However, HRV parameters were different in controls and HD patients (SDNN controls 34.0 [14.0] ms, HD patients 15.5 [14.8] ms, *p* < 0.01). This finding was not restricted to patients with diabetes mellitus (diab), although diabetes is an important cause of autonomous dysfunction (SDNN, diab 13.0 [14.0] ms, non-diab 18.0 [15.3] ms, *p* = 0.8). LF and HF were reduced by the same magnitude to 1/3 of those in controls. Patients suffered from chronic inflammation (CRP 9.4 [12.9] mg/l, controls 1.6 [2.4] mg/l, *p* < 0.001) and expanded proinflammatory monocyte subpopulations (CD14++/CD16+ cells: patients 41 [27]/μl, controls 24 [18]/μl, *p* < 0.01). ECG parameters did not correlate with inflammation in patients, but monocyte ACh receptor expression was enhanced, indicating potentially elevated responsiveness of this cell type to parasympathetic regulation.

**Conclusions:**

HD patients have strongly impaired HRV. Chronic inflammation is not related to autonomous dysfunction, although monocytes express the ACh receptor at enhanced density making them potentially more sensitive to parasympathetic effects.

**Trial registration:**

This study was listed with ClinicalTrials.gov (NCT00878033).

**Electronic supplementary material:**

The online version of this article (doi:10.1186/s12872-016-0385-1) contains supplementary material, which is available to authorized users.

## Background

**Fig. 1:**
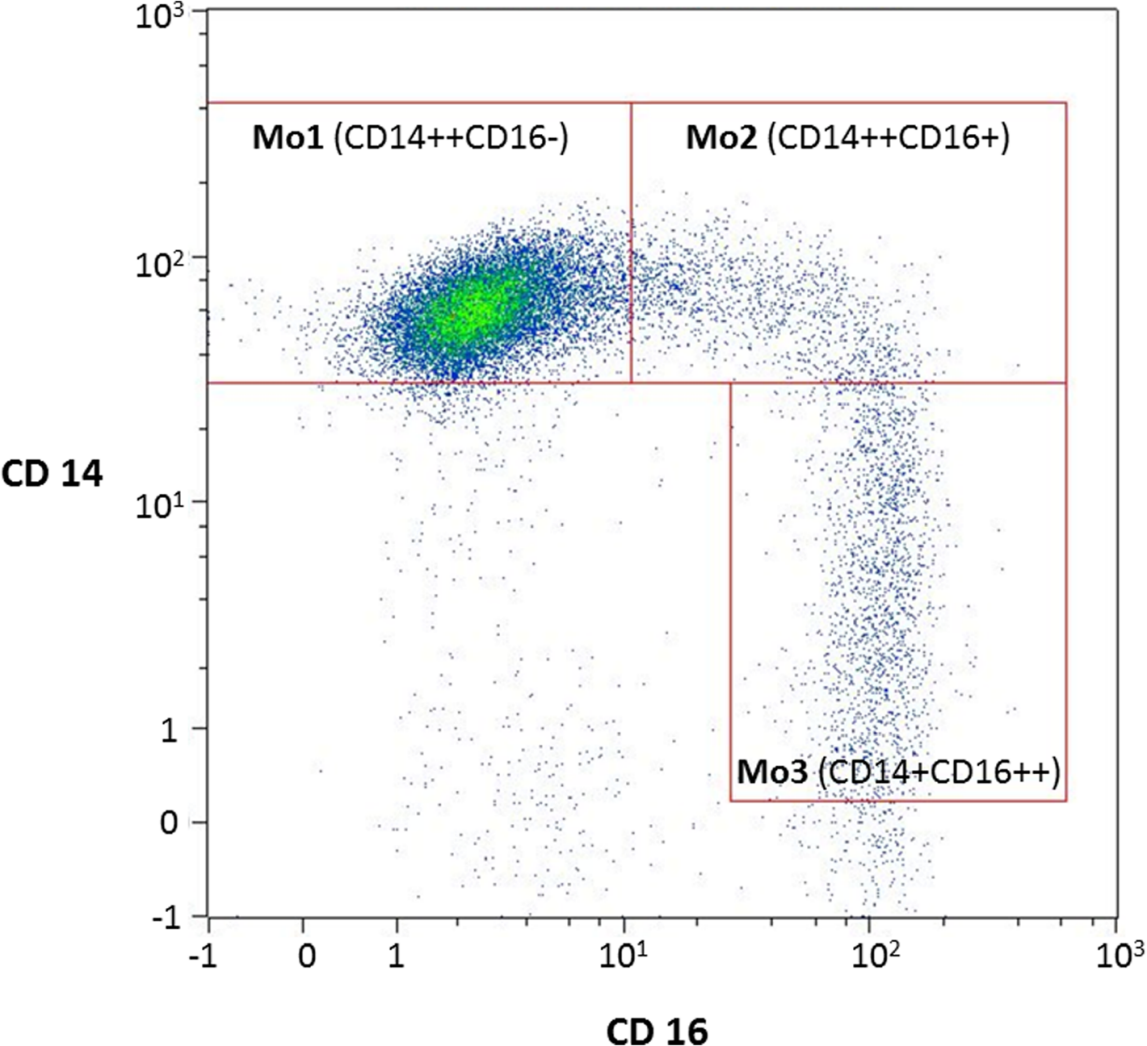
Monocyte subpopulations in a flow cytometry read-out. All cells showed monocyte characteristics, however, the populations differ in their expression density of CD14 and CD16. The Mo2 cell type is linked to enhanced inflammation and adverse clinical outcome in dialysis patients [4]

Cardiovascular mortality in end-stage renal disease (ESRD) patients is high while classical (Framingham) risk factors are insufficient to fully explain the event rates [1]. The high level of inflammation was established as an additional risk factor [2, 3] and may be a metabolic consequence of uremia. Several immune-active proteins are eliminated through the kidneys and retained in chronic renal failure. Nevertheless, the pathogenesis of inflammation in renal failure is not fully understood and monocytes of the peripheral blood are important contributors. Sizes of several functionally and morphologically defined subpopulations differ from healthy individuals with elevated circulating numbers of pro-inflammatory cells. Monocyte subpopulation numbers are predictive for cardiovascular event rate and all-cause mortality [4]. Three functionally distinct cell populations, Mo1, Mo2 and Mo3 (Fig. 1) can be distinguished, differing in expression density of the endotoxin receptor CD14 and the immunoglobulin Fc segment receptor CD16. Mo2 have particular pro-inflammatory properties and are linked to adverse outcome in dialysis patients [4].

The autonomic nervous system might directly influence activation of monocytes through ß-adrenoceptors or receptors for acetylcholine (AchR). Stimulation of ß-adrenoceptors downregulates the production of cytokines such as IL-12, IL-18 [5] and MIP-1 [6], and adhesion molecules ICAM-1, CD40 and CD14 [7] while other secretion products (IL-8,[8]) or functional enzymes (matrix metalloproteinases,[9]) are upregulated. Stimulation of the AchR inhibits the pro-inflammatory response of macrophages [10] and monocytes [11]. This suggests that the function of monocytes is tightly controlled by the autonomic nervous system.

Renal failure strongly influences the autonomic nervous system even in early stages of chronic kidney disease (CKD). Several studies documented a sympathetic overstimulation (reviewed in [12]) in dialysis patients that reduces the expression of adrenoceptors on target cells in addition to uremia that reduces the expression of ß-adrenoceptors [13] itself. The function of the parasympathetic axis is less well studied. Work from the 1980s indicated parasympathetic dysfunction, however, these studies used only classical cardiovascular reflex mechanisms (baroreceptor reflex, valsalva-technique) [14].

Few studies addressed autonomous nervous dysfunction in chronic dialysis patients by assessing the variability of heart frequency [7, 15, 16]. In general, heart rate variability (HRV) is sensitive to extracorporeal treatment. The reduction of intravascular volume by ultrafiltration modulates the frequency domain parameters low frequency power (LF) and high-frequency (HF) power [17] which might be a consequence of sympathetic counterregulation of volume depletion. Recently, it was shown in CKD patients, that HRV is a predictor of adverse outcomes [18, 19] and is associated with left ventricular hypertrophy [20, 21].

Effects of the autonomic nerve system on cardiac function can be diagnosed using time-domain (SDNN and pNN50), frequency-domain (HF and LF) and nonlinear (erratic) HRV [22]. Recently, the interpretation of LF and LF/HF ratio as indices of sympathetic cardiac control and autonomic balance, respectively, has been challenged and it was suggested that the HRV power spectrum, including its LF component, is mainly determined by the parasympathetic system. LF power may rather provide an index of baroreflex function [23].

This study addresses the role of autonomous dysregulation for inflammation in ESRD. Autonomous neuropathy is common in diabetic patients [24, 25] and the prevalence of diabetes mellitus is high in German dialysis populations [26]. Therefore, we included a comparison group with diabetic hemodialysis patients to evaluate autonomous neuropathy in relation to the presence of diabetes mellitus. We investigated the susceptibility of different cell populations to signals mediated by acetylcholine and the relation between autonomous nerve dysfunction measured by ECG analysis and the systemic inflammation measured by humoral and cellular markers.

## Methods

### Study subjects

We studied 30 ESRD patients on hemodialysis (HD) and 15 healthy control individuals. Among the patients, 15 were diabetic (self-reported diabetes mellitus and antidiabetic oral (20 %) or parenteral (80 %) medication). All subjects were studied cross-sectionally at one visit. The patients were selected from a single German non-profit out-patient dialysis center with ESRD for at least 3 months and an age above 18 years with stable sinus rhythm. Exclusion criteria were clinical signs of acute infection, active malignancy, CRP above 50 mg/L, presence of a cardiac pacemaker, atrial fibrillation, heart transplantation and immunosuppressive medication. Healthy controls had no known history of renal or heart disease and values within the normal range for serum creatinine, urea, CRP, BNP, pH and bicarbonate.

### Study procedures

Patients and control individuals were assessed for age, body height, body weight and waist-to-hip ratio. A resting blood pressure was determined using an automated oscillometric system (dinamap, GE Systems). Patients were interviewed for dialysis vintage and residual diuresis and data were consolidated using the patient’s files. Ultrafiltration volume was taken from the dialysis monitor readings and the ultrafiltration rate was calculated from the ultrafiltration volume and the effective dialysis time.

The following parameters were measured by routine laboratory methods in an accredited diagnostic laboratory from blood samples drawn before the first dialysis of the week: creatinine, urea, cholesterol, HbA1c, CRP, interleukin-6 (IL-6), Bicarbonate, brain natriuretic peptide (BNP) and an automated blood differential count. HRV analysis was performed once within 2 weeks before to 2 weeks after the blood samples.

For analysis of monocytes, lymphocytes and B-cells, lithium heparinized whole blood was washed twice in phosphate-buffered saline/2 mM EDTA/0.5 % bovine serum albumin/0.07 % sodium azide. Monocyte staining was performed using anti-CD86 PeCy5 (clone IT2.2, Beckman Coulter, Krefeld, Germany), −CD14 PeCy7 (clone MPhi9, Becton Dickinson, Heidelberg, Germany), −CD143 FITC (clone 9B9, AbD Serotec, Düsseldorf, Germany), −CD16 APC (clone CB16, eBioscience), −CD19 (clone SJ24C1, eBioscience) and -CD15 eF450 (clone HI98, ebioscience) antibodies. Lymphocyte and B-cell staining comprised anti-CD3 FITC (clone BW264/56, Miltenyi Biotec, Bergisch-Gladbach, Germany), −CD56 V450 (clone B159, BD, Heidelberg, Germany), −CD19 APCeF780 (clone HIB19, ebioscience). For identification of AChR on peripheral blood leucocytes nicotinic acetylcholine receptor alpha subunit (clone G10, Biozol, Eching, Germany) was used. Specificity of this antibody has been evaluated earlier [27–29]. The FACS staining protocols included fluorescence-minus-one-controls. To take unspecific binding by IgG into account, isotype controls were used to allow for better correction of gating. Specifity of staining was confirmed by use of isotypic IgG1 staining (Biozol, Eching, Germany). R-Phycoerythrin labelling of AChR as well as the corresponding Isotype control (IgG) was done using the LYNX RAPID RPE Antibody Conjugation Kit® (Abd Serotec, Düsseldorf, Germany).

**Fig. 2:**
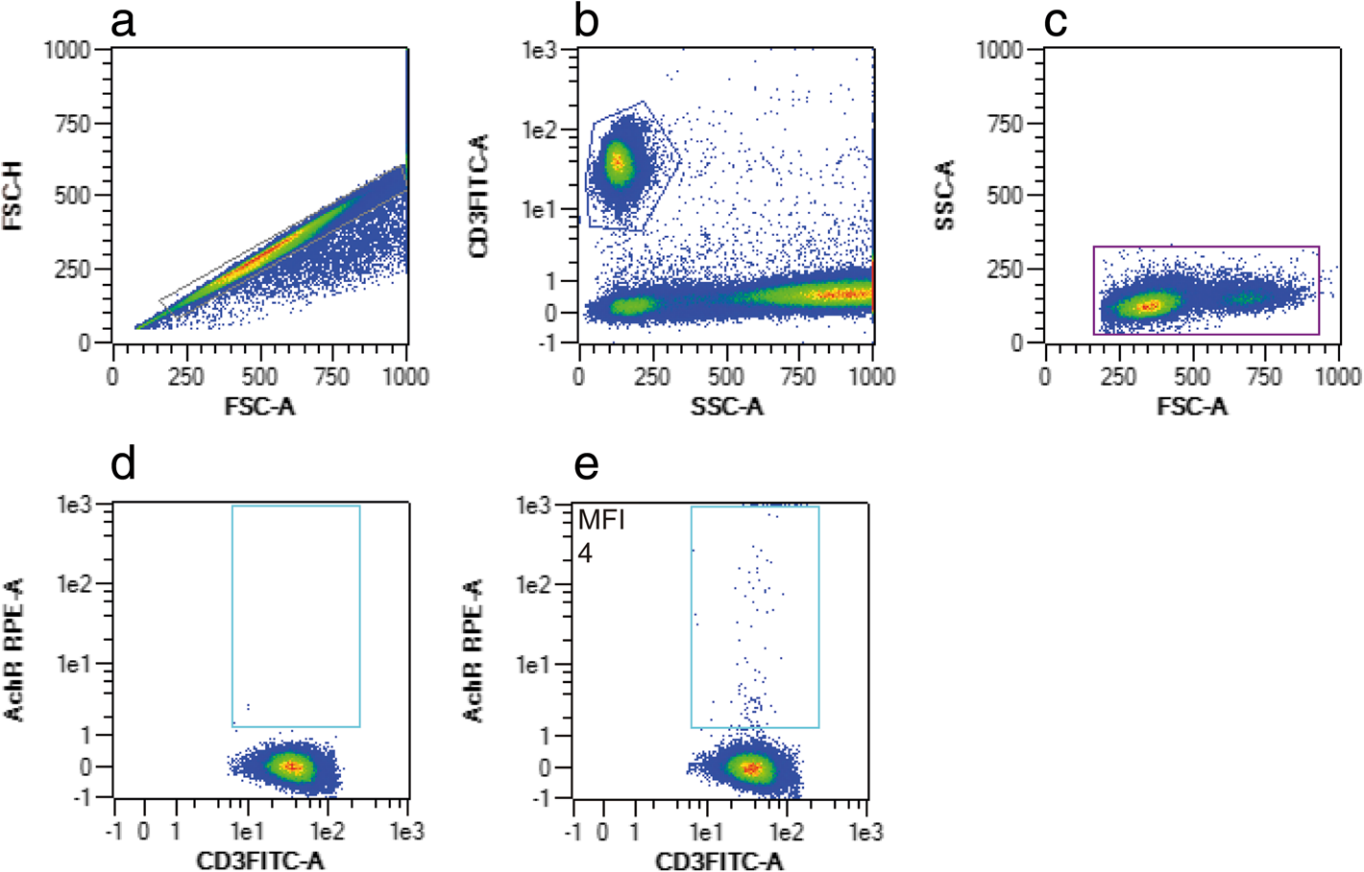
Flow cytometric gating strategy of CD3-positive T-cells expressing nicotinic acetylcholine receptor (AchR). After exclusion of doublets (**a**), lymphocytes were characterized by anti-CD3 staining (**b**) and by back-gating in a FSC/SSC plot (**c**). Plots **d** and **e** are representative of isotype control and the corresponding sample staining positive for CD3 + AchR+

**Fig. 3:**
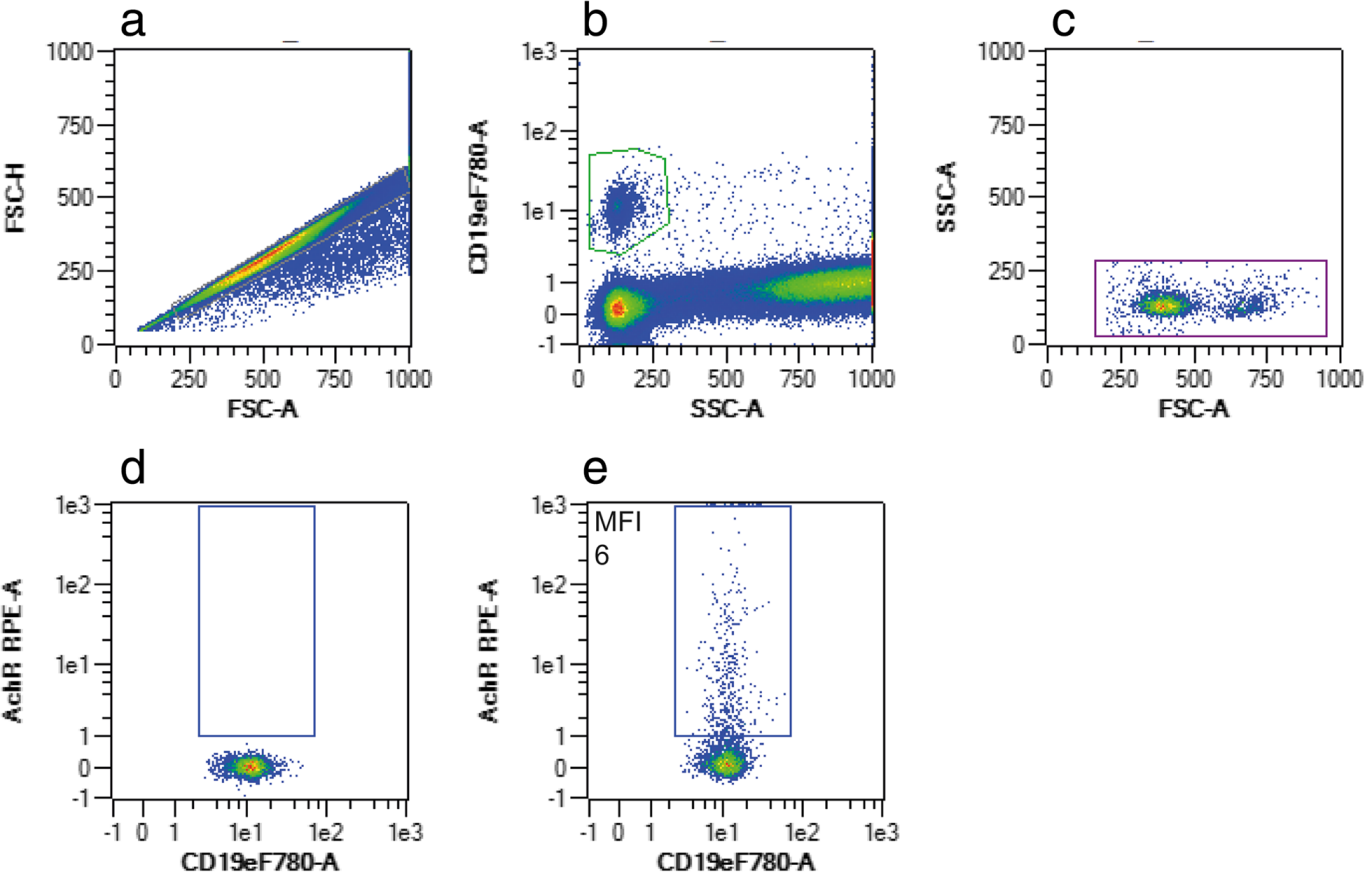
Flow cytometric gating strategy of CD19-positive B-cells expressing nicotinic acetylcholine receptor (AchR). After exclusion of doublets (**a**), B-cells were characterized by anti-CD19 staining (**b**) and by back-gating in a FSC/SSC plot (**c**). Plots **d** and **e** are representative of isotype control and the corresponding sample staining positive for CD19 + AchR+

**Fig. 4:**
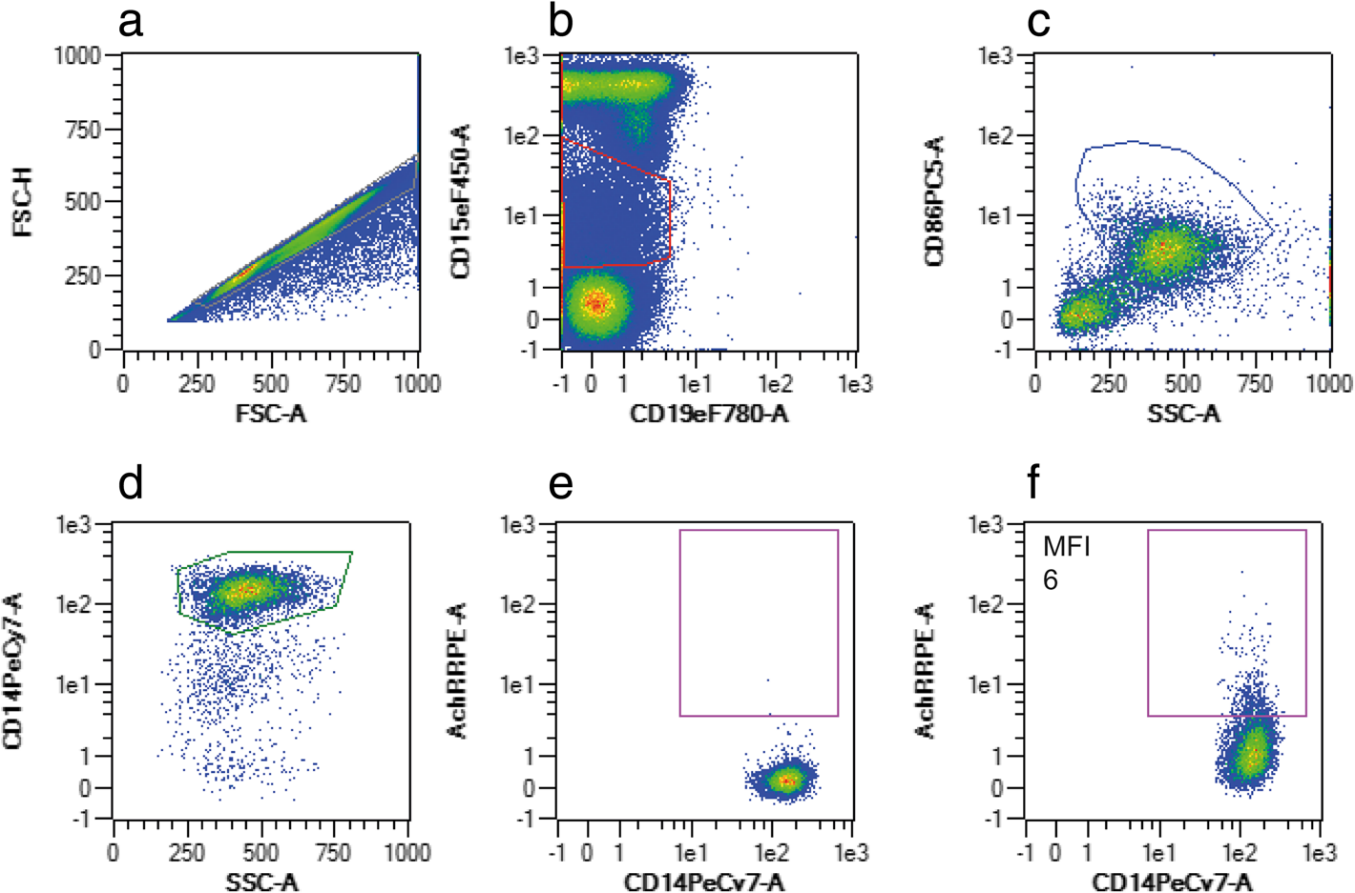
Flow cytometric gating strategy of CD14-positive monocytes expressing nicotinic acetylcholine receptor (AchR). After exclusion of doublets (**a**), CD15++ granulocytes and CD19+ B-cells were excluded (**b**). Monocytes were characterized by anti-CD86- (**c**) and by anti-CD14-staining (**d**). Plots **e** and **f** are representative of isotype control and the corresponding sample staining positive for CD14 + AchR+

By flow cytometry (MACSQuant, Miltenyi Biotech, Bergisch-Gladbach, Germany), monocytes were defined as CD15-CD19-CD86+. Subpopulations were divided according to the expression of CD14 and CD16 (CD14++CD16-: Mo1, CD14++CD16+: Mo2, CD14 + CD16+: Mo3). Absolute cell numbers were calculated from the relative numbers detected by flow cytometry and the absolute monocyte numbers detected by automated blood differential count. Expression density of ACE (CD143) was analyzed by measuring the median fluorescence intensity on Mo2 cells. Expression density of the AChR was analyzed by measuring the median fluorescence intensity on all cells that were positive for either CD14, CD3, or CD19. The gating strategy included doublets exclusion and characterisation of leukocyte subsets by staining with specific markers, i.e. anti-CD3 for T-cell, anti-CD19 for B-cell and anti-CD86 for monocyte gating (Figs. 2, 3 and 4).

A 12-lead resting 20-min ECG was recorded on a CardioControl Working Station (Welch Allyn, Delft, the Netherlands) after a resting period in the supine position of ≥20 min. Throughout the ECG, subjects were asked to breathe at 15 breaths/min guided by a visual metronome to standardize the influence of the respiratory rate on spectral HRV parameters. It is known that naturally occurring fluid shifts in dialysis patients within the interdialytic interval may influence ECG recordings. For example, it has been demonstrated that the LF/HF ratio changes over time when measured before, during, or after a dialysis session [16]. Therefore, ECG recordings were executed after the hemodialysis sessions with all patients being at dry weight. For the same reason, 20-min ECG-duration was chosen instead of conventional 24 h recordings. Although the use of time domain parameters has been recommended by the Task Force of The European Society of Cardiology and The North American Society of Pacing and Electrophysiology for long-term ECGs only [22], several studies found good ability of SDNN for prediction of mortality also from short-segment ECGs [30, 31].

All ECGs were processed by the Modular ECG Analysis System (MEANS) [32] to automatically obtain the location and type of the QRS complexes of the 20-min ECG. A visual check was performed by a medical student which was supervised by a cardiologist. For further analyses, HRV derived from the best 5-min segment of the 20-min ECG was used according to the following quality criteria: proportion of abnormal beats; mean and variance stationarity score of the tachogram. Information on QRS complexes was used to compute standard time and frequency domain parameters of HRV for 5-min segments of the ECG according to the current guidelines for the analysis of HRV [22]. Artefacts and ectopic beats were replaced by interpolated normal sinus beats. We used the standard deviation of normal intervals (SDNN in ms) and the number of pairs of adjacent NN intervals differing by more than 50 ms divided by the total number of all NN intervals (pNN50 in %) – both time domain parameters – and the frequency domain parameters low frequency power (LF) (0.04 to <0.15 Hz), high-frequency (HF) power (0.15–0.4 Hz), the ratio of LF to HF (LF/HF) and very low frequency (VLF) power (<0 · 04 Hz). To calculate frequency domain parameters, tachograms of RR intervals were adjusted for linear trends, tapered and zero-padded and a Fast Fourier transformation was employed.

### Statistical analysis

Data were analysed using the IBM SPSS Statistics 22 software package (IBM Corporation 2013, Somer, NY, USA). Univariate analyses of metric outcomes were performed with the Kruskal-Wallis test with Dunn-Bonferroni post hoc test. Contingency tables were tested using Chi^2^ tests. Results were adjusted for multiple testing. Data are presented as median [Inter quartile range (IQR)].

We further calculated gender-, age- and heart rate-adjusted means (±95 % confidence interval (CI)) of HRV parameters by diabetic HD, nondiabetic HD and healthy controls using linear regression models. The F-test was used to test the difference in adjusted means of HRV between diabetic HD, nondiabetic HD and healthy controls. Multifactorial analyses were undertaken using SAS 9.3 (SAS Institute, Cary, NC, USA).

Sample size estimation was based on the putative difference of SDNN between ESRD patients and healthy controls. According to previous findings a difference of 66 ms was established [33]. The study should be able to show this difference at a sample size of ≥ 6 in each observation group (controls, diabetic and non-diabetic HD patients) with a power of 0.95 and an α error probability of 0.05. For sample size estimation, G*Power Version 3.1.9.2 [34, 35] was used.

## Results

### Study subjects

Causes of ESRD were: 15 patients: diabetic nephropathy, 4: glomerulonephritis, 3: nephrosclerosis, 3: interstitial disease, 3: unknown origin, 1: polycystic kidney disease, 1: thrombotic microangiopathy. One diabetic patient had to be excluded from analysis as his ECG registrations could not be evaluated for technical reasons. We excluded one further patient from HRV analyses because of erratic heart rhythm, which was detected by poincaré plot analysis [36]. Subject demographics are listed in Table 1. All patients were treated thrice weekly for at least 4 h with similar single-use synthetic high-flux dialyzers and ultrapure dialysate and heparinate anticoagulation. Further treatment parameters and cardiovascular assessments are reported in Table 2.

**Table 1:** Demographic and anthropomorphometric information on study participants

	Controls	Diabetic HD	Nondiabetic HD	All HD
N	15	14	14	28
Age (years)	58.0 [13.0]	70.5 [9.75]***	62.0 [17.25]	68.0 [14.75]
Gender (w/m)	10/5	5/9	7/7	12/16
Dialysis vintage (years)	n/a	3.5 [3.2]	5.2 [5.9]	4.3 [5.8]
BMI (kg/m^2^)	26.8 [5.2]	30.1 [6.0]	24.5 [6.4]	27.5 [8.2]
Waist-to-hip-ratio (WTH)	1.0 [0.28]	1.02 [0.14]	0.97 [0.18]	1.0 [0.16]
Blood pressure s/d (mmHg)	130.0 [10.0]/83.0 [15.0]	121.5 [42.8]/70.5 [28.0]**	120.5 [44.8]/68.5 [13.3]**	120.5 [39.0]/68.5 [23.5]

** = *p* < 0.01 vs. controls; *** = *p* < 0.001 vs. controls; by nonparametric Kruskal-Wallis and Dunn’s test. Statistical tests were done for the three groups controls, diabetic and nondiabetic patients. The column “all HD” remained untested

**Table 2:** Treatment associated parameters of the dialysis patients

	Diabetic HD	Nondiabetic HD	All HD
Patients with diuresis < 200 ml/d (n)	5/14	8/14	13/28
Residual diuresis (ml/d)	400 [563]	100 [525]	250 [500]
Ultrafiltration volume (per session)	3000 [1000]	1800 [2750]	2750 [1600]
Ultrafiltration rate (ml/h)	506 [427]	416 [452]	431 [425]
Reduction in body weight (kg)	2.3 [0.7]	1.6 [2.1]	2.25 [1.47]
Predialysis blood pressure (mmHg)	121.5 [42.8]/70.5 [28.0]	120.5 [44.8]/68.5 [13.3]	120.5 [39.0]/68.5 [23.5]
Predialysis heart rate (/min)	74.5 [13.5]	72.0 [21.3]	74 [15.8]
Postdialysis blood pressure (mmHg)	126.5 [40.8]/67.0 [17.8]	115.0 [35.5]/66.0 [16.0]	120.0 [43.0]/66.0 [15.0]
Postdialysis heart rate (/min)	73 [14]	76 [21.3]	76 [16.3]

There were no statistically significant differences between diabetic and nondiabetic dialysis patients. [Chi^2^-test, Mann–Whitney test]

### Routine laboratory data

As expected, there was a large difference in creatinine and urea values. However, also CRP and IL-6 values were significantly higher in dialysis patients. Furthermore, a large range was noted for BNP values but none of the patients had clinical signs of heart failure. Of note, the diabetic dialysis patients had a balanced glucose metabolism and HbA1c was within the goal range in most of the participants. All results are listed in Table 3.

**Table 3:** Routine laboratory results

	Controls *N* = 15	Diabetic HD *N* = 14	Nondiabetic HD *N* = 14	All HD *N* = 28
Creatinine (μmol/l)	71.0 [23.0]	531.0 [298.0]**	887.0 [246.5]***	726.5 [400.5]
Urea (mmol/l)	5.0 [1.5]	18.2 [8.6]***	17.9 [9.3]***	17.9 [8.7]
Cholesterol (mmol/l)	4.9 [1.3]	4.6 [2.2]	4.7 [1.7]	4.6 [1.7]
HbA1c (%)	5.5 [0.4]	5.9 [2.1]	5.5 [0.5]	5.6 [0.8]
CRP (mg/l)	1.6 [2.4]	12.6 [9.6]***	4.8 [13.0]	9.4 [12.9]
IL-6 (pg/ml)	1.1 [1.1]	9.7 [12.8]***	4.7 [6.7]**	6.9 [12.6]
Bicarbonate (mmol/l)	25.9 [3.5]	22.8 [1.7]*	22.2 [3.9]**	22.3 [2.6]
BNP (pg/ml)	26.0 [35.0]	287.0 [695.8]***	113.0 [420.8]**	262.0 [401.3]

In some individuals, CRP levels were reported by the lab as < 1 mg/l and IL-6 levels as < 0.5 pg/ml. These data points were entered as 0.5 mg/l and 0.3 pg/ml, respectively.

* = *p* < 0.05 vs. controls; ** = *p* < 0.01 vs. controls; *** = *p* < 0.001 vs. controls by Kruskal-Wallis and Dunn’s test. Statistical tests were done for the three groups controls, diabetic and nondiabetic patients. The column “all HD” remained untested

### ECG analysis

Time-domain parameters (SDNN, pNN50) and frequency-domain parameters (VLF, LF, HF as well as the LF/HF ratio) are reported in Table 4. There were marked differences in all ECG parameters between patients and healthy controls. However, diabetic and non-diabetic hemodialysis patients did not differ with regard to the ECG parameters. After correction for age and gender, differences in heart rate, SDNN and VLF remained significant (Additional file 1: Table S1). Also, after additional correction for heart rate, results were unchanged (Table 5).

**Table 4:** Raw means ± SD of ECG analysis parameters in healthy controls and dialysis patients

	Controls (*N* = 15)	Diabetic HD (*N* = 14)	Nondiabetic HD (*N* = 14)	All HD (*N* = 28)
Heart rate (/min)	69.0 [12.0]	78.0 [14.0]*	78.5 [28.3]	78.5 [19.5]
SDNN (ms)	34.0 [14.0]	13.0 [14.0]**	18.0 [15.3]**	15.5 [14.8]
pNN50 (%)	3.0 [8.0]	0.5 [1.3]	0.0 [1.3]*	0.0 [1.0]
VLF (s^2^ x10^−3^)	0.72 [0.87]	0.12 [0.21]**	0.20 [0.35]**	0.12 [0.29]
LF (s^2^ x10^−3^)	0.39 [0.29]	0.035 [0.21]**	0.076 [0.20]**	0.052 [0.18]
HF (s^2^ x10^−3^)	0.27 [0.22]	0.08 [0.1]**	0.06 [0.11]**	0.07 [0.09]
LF/HF	1.1 [1.3]	0.8 [1.0]	0.7 [2.2]	0.8 [1.2]

* = *p* < 0.05 vs. controls; ** = *p* < 0.01 vs. controls; *** = *p* < 0.001 vs. controls; by nonparametric Kruskal-Wallis and Dunn’s test. Statistical tests were done for the three groups controls, diabetic and nondiabetic patients. The column “all HD” remained untested

**Table 5:** Gender-, age and heart rate adjusted means (95 % CI) of ECG analysis parameters in healthy controls and dialysis patients

	Controls (*N* = 15)	Diabetic HD (*N* = 14)	Nondiabetic HD (*N* = 14)
Heart rate (/min)	65.37 (57.50–73.25)	79.89 (71.69–88.10)	78.79 (71.89–85.70)*
SDNN^a^ (ms)	31.11 (22.53–42.96)	17.03 (12.34–23.49)	17.75 (13.55–23.25)*
pNN50 (%)	6.13 (0–12.57)	5.36 (0–11.78)	2.28 (0–7.67)
VLF^a^ (s^2^ x10^−3^)	0.57 (0.30–1.08)	0.14 (0.07–0.27)*	0.20 (0.12–0.34)
LF^a^ (s^2^ x10^−3^)	0.32 (0.13–0.76)	0.08 (0.03–0.19)	0.09 (0.04–0.18)
HF^a^ (s^2^ x10^−3^)	0.27 (0.14–0.52)	0.10 (0.05–0.20)	0.07 (0.04–0.12)*
LF/HF^a^	1.19 (0.65–2.17)	0.76 (0.41–1.38)	1.27 (0.77–2.11)

* = *p* < 0.05 vs. controls; 95 % CI: 95 % Confidence Interval.

^a^Because of skewness of the distribution of the HRV parameters we calculated geometric means (± 95 % CI)

### Monocyte parameters

Results of total monocyte number, monocyte subpopulation quantification (Mo1, Mo2, Mo3) and monocyte expression density of CD143 (angiotensin converting enzyme) are reported in Table 6. Mo2 and Mo3 populations were expanded in diabetic and non-diabetic dialysis patients. There was a higher expression of CD143 on Mo2 and Mo3 cells in dialysis patients compared to healthy individuals, albeit it did not reach statistical significance.

**Table 6:** Monocyte and flow cytometry parameters

	Controls	Diabetic HD	Nondiabetic HD	All HD
Monocytes (/μl)	480 [210]	600 [430]	535 [350]	565 [370]
Mo1 (/μl)	374 [187]	441 [249]	354 [290]	428 [260]
Mo2 (/μl)	24 [18]	40 [59]	41 [21]*	41 [27]
Mo3 (/μl)	76 [43]	121 [104]	126 [69]*	126 [90]
CD143+/Mo1 (MFI)	1.1 [1.8]	1.5 [6.0]	2.1 [8.6]	1.8 [7.5]
CD143+/Mo2 (MFI)	2.2 [1.8]	2.9 [7.1]	4.3 [10.4]	3.7 [8.0]
CD143+/Mo3 (MFI)	2.7 [1.4]	4.2 [5.0]	5.0 [5.6]**	4.3 [5.0]

* = *p* < 0.05 vs. controls; ** = *p* < 0.01 vs. controls by Kruskal-Wallis and Dunn’s test. Statistical tests were done for the three groups controls, diabetic and non-diabetic patients. The column “all HD” remained untested. *MFI:* Mean fluorescence intensity

Table 7 reports AChR density on monocytes (as defined by the coexpression of CD86 and CD14), B-lymphocytes (CD19+) and T-lymphocytes (CD3+). Monocytes showed a higher expression of the AChR in dialysis patients compared to controls. In contrast, the expression of AChR was not different on B- or T-lymphocytes.

**Table 7:** Expression density of acetylcholine receptor (AChR) on CD14+ monocytes, CD3+ T-cells, and CD19+ B-cells

	Controls	Diabetic HD	Nondiabetic HD	All HD
AChR/CD14+ Monocytes (MFI)	7.0 [1.8]	15.4 [6.9]*	15.3 [3.3]**	15.4 [5.2]
AChR/CD3+ T-Lymphocytes (MFI)	14.3 [8.3]	15.9 [5.3]	11.7 [5.3]	13.1 [5.9]
AChR/CD19+ B- Lymphocytes (MFI)	10.8 [3.0]	13.3 [5.3]	14.3 [8.4]	14.2 [5.7]

* = *p* < 0.05 vs. controls; **p < 0.01 vs. controls by Kruskal-Wallis and Dunn’s test. Statistical tests were done for the three groups controls, diabetic and nondiabetic patients. The column “all HD” remained untested. *MFI:* Mean fluorescence intensity

### Correlation analysis

Dialysis treatment parameters as well as hemodynamic parameters before and after a dialysis session were not related to the ECG analysis results. Also, ECG-derived markers of autonomous regulation were only weakly correlated to the monocyte subpopulation measurements (Table 8).

**Table 8:** Linear regression coefficients between ECG parameters and monocyte subpopulation counts in dialysis patients

	Heart rate (/min)	SDNN (ms)	VLF (x10^−3^/s)	LF (x10^−3^/s)	HF (x10^−3^/s)
Mo1 (/μl)	0.09*	0.07	0.07	0.02	0.05
Mo2 (/μl)	0.10*	0.14*	0.12	0.03	0.06
Mo3 (/μl)	0.11*	0.23*	0.06	0.02	0.15*

* = *p* < 0.05

## Discussion

### Heart rate variability

ECG analysis revealed strong signs of autonomous dysfunction. In both diabetic and non-diabetic dialysis patients the time-domain parameters of HRV analysis were severely impaired. This means that there is strong autonomous neuropathy that seems to be independent from the presence of diabetes mellitus.

In addition, the frequency-domain parameters were also strongly impaired. Customarily, LF was thought to reflect sympathetic influence on cardiac function whereas HF variation should be a marker of parasympathetic influences. Therefore, the ratio of LF to HF was considered to reflect the balance between sympathetic and vagal activity. Recently, this interpretation has been challenged and it was suggested that the HRV power spectrum is mainly determined by the parasympathetic system [37]. LF power may rather provide an index of baroreflex function [23].

Earlier studies showed that patients with chronic renal failure have a strong sympathetic overactivity [12] but little is known about parasympathetic activity in these individuals. Our data indicate that parasympathetic activation seems to be strongly diminished. Most likely, our findings result from the unexpectedly strong autonomous neuropathy, which renders the frequency-domain analysis inconclusive. Of note, the LF/HF ratio was not different between dialysis patients and controls, not even when analyzing only diabetic dialysis patients. This supports the notion that the global changes by renal failure disable the frequency-domain analysis. However, it has been revealed in a validation study with pharmacological blocking that LF/HF ratio is only weakly correlated with robust measures of autonomic tone [38]. Therefore, these results should be interpreted with caution. Additionally, the use of frequency-domain parameters has been criticized recently. Time-domain parameters are considered more robust since they can be estimated with smaller bias and considerably smaller variability than frequency domain parameters [39].

The conclusion that the specific balance between sympathetic and vagal tone in hemodialysis patients cannot be reliably measured by ECG analysis while reduced heart rate variability is a useful measure of autonomic dysfunction in general is compatible with recent publications on this topic. Kurata et al. [33] found high catecholamine levels as signs of sympathetic overactivation together with reduced heart rate variability (low SDNN).

A study by Fukuta et al. addressed heart rate variability in 120 chronic hemodialysis patients with similar age and dialysis vintage as in our patients [40]. They used a different ECG analysis technique based on 24 h Holter monitoring on the day between dialysis treatments. Therefore, the absolute measurements for time- and frequency-domain parameters cannot be compared to our data. Nevertheless, their findings are important to our study, since they demonstrated impaired R-R variation and low values for LF and HF variation in their patients. Furthermore, patients with the strongest impairment in heart rate variability were more likely to die from cardiovascular causes within the observation period of 26 months.

A recent study [41] showed that the initiation of hemodialysis therapy in advanced stage 4–5 CKD patients improves several measures of heart rate variability. Thus, uremic intoxication in general seems to play an important role.

The lack of echocardiographic data might appear as a significant limitation. However, none of the patients of the study had any sign of heart failure. Additionally, BNP measures in dialysis patients with echocardiographically diagnosed heart failure were more than 5 times higher than in our population [42] and BNP levels in our population are within a range that was identified as beneficial in a prospective 2-year mortality analysis [43].

### Inflammation

Preliminary data from Psychari et al. [44] suggested a relation between markers of autonomous dysfunction and systemic inflammation. They demonstrated a correlation between SDNN values and IL-6 plasma levels in patients with CKD 3–4. In addition, the expression of adrenoceptors and AchRs on monocytes and the multiple effects that sympathetic [5, 6, 8, 9, 45] as well as parasympathetic [11] stimulation has on these cells, further supports this hypothesis. We could confirm earlier data on chronic inflammation and its extent in dialysis patients [4, 46, 47]. The inflammation markers CRP and IL-6 were greatly enhanced in both patient groups and the pro-inflammatory monocyte subpopulations were expanded. Furthermore, the expression of the angiotensin converting enzyme ACE on the surface of monocytes tended to be higher in dialysis patients compared to healthy controls. This also confirms earlier findings on subpopulation alterations and their enhanced expression of ACE [48–50]. Since ACE expression on Mo2 cells is related to adverse outcome and enhanced cardiovascular disease in dialysis patients, these cells are thought to play a causal role in the progression of atherosclerosis. However, our study is limited by the different cardiovascular medication in cases and controls. The high burden of cardiovascular disease in ESRD makes it unlikely to recruit dialysis patients without cardiovascular medication. Therefore, confounding immunomodulatory effects can not be entirely ruled out. Also, heparinate anticoagulation has been discussed to posess anti-inflammatory effects. There is some low level clinical evidence in asthma patients with mixed results. Effects in other inflammatory diseases have been equivocal [51, 52]. However, it cannot be entirely ruled out that heparinate anticoagulation might influence the immune system, especially in vitro and in supraphysiological doses higher than in the dialysis circuit.

Interestingly, expression of the AchR is significantly elevated in CD14+ Monocytes in ESRD patients compared to controls. This is not the case for other cell populations such as CD3+ T-Lymphocytes and CD19+ B-Lymphocytes. It might appear as a limitation of our study, that antibody specificity was not tested with alternative approaches such as knockout cells/mice in addition to previous investigations [27–29].

However, our analysis showed that there was no relation between markers of autonomous dysfunction and the studied markers of systemic and cellular inflammation. Additionally, we have at least no hint, that autonomic nerve dysfunction might relevantly influence monocyte subpopulation composition in dialysis patients. Although negative, this is an important clarification in the scientific debate. Monocyte subpopulation composition may be influenced by several conditions in chronic renal failure. The altered balance of sympathetic and parasympathetic tone can now be excluded as a major regulator of monocyte differentiation.

## Conclusions

In conclusion, dialysis patients show signs of autonomic derangement in their heart rate variability. Because their heart rate is elevated, they are likely to have a sympathovagal balance that is shifted towards the sympathetic side. A possible explanation of the low amount of heart rate variability is a lowered baroreflex sensitivity. However, such an explanation remains speculative because baroreflex sensitivity was not measured in our study. Further studies that include baroreflex measurements are needed to investigate this.

Second, there is no significant correlation to the studied markers of cellular and humoral inflammation. However, monocyte function might be associated to sympathetic tone as indicated by the expression density of the acetylcholine receptor exclusively on these cells. Further studies including functional assays are needed to further investigate this possibility.

## Additional file

Additional file 1: Table S1. Gender- and age-adjusted means ± SE of ECG analysis parameters in healthy controls and dialysis patients. (DOCX 13 kb)

## Abbreviations

ACh: Acetylcholine
AChR: Receptors for acetylcholine
CRP: C-reactive protein
ECG: Electrocardiogram
ESRD: End-stage renal disease
HD: Hemodialysis
HF: High-frequency variation of RR intervals
HRV: Heart rate variability
IL: Interleukin
LF: Low-frequency variation of RR intervals
pNN50: Percentage of pairs of adjacent RR intervals differing by >50 ms
SDNN: Standard deviation of the RR intervals

## References

[1] Cheung AK, Sarnak MJ, Yan G, Dwyer JT, Heyka RJ, Rocco MV (2000). Atherosclerotic cardiovascular disease risks in chronic hemodialysis patients. Kidney Int.

[2] Ridker PM, Rifai N, Rose L, Buring JE, Cook NR (2002). Comparison of C-reactive protein and low-density lipoprotein cholesterol levels in the prediction of first cardiovascular events. N Engl J Med.

[3] Zimmermann J, Herrlinger S, Pruy A, Metzger T, Wanner C (1999). Inflammation enhances cardiovascular risk and mortality in hemodialysis patients. Kidney Int.

[4] Heine GH, Ulrich C, Seibert E, Seiler S, Marell J, Reichart B (2008). CD14(++)CD16+ monocytes but not total monocyte numbers predict cardiovascular events in dialysis patients. Kidney Int.

[5] Mizuno K, Takahashi HK, Iwagaki H, Katsuno G, Kamurul HASM, Ohtani S (2005). Beta2-adrenergic receptor stimulation inhibits LPS-induced IL-18 and IL-12 production in monocytes. Immunol Lett.

[6] Li C-Y, Chou T-C, Lee C-H, Tsai C-S, Loh S-H, Wong C-S (2003). Adrenaline inhibits lipopolysaccharide-induced macrophage inflammatory protein-1 alpha in human monocytes: the role of beta-adrenergic receptors. Anesth Analg.

[7] Axelrod S, Lishner M, Oz O, Bernheim J, Ravid M (1987). Spectral analysis of fluctuations in heart rate: an objective evaluation of autonomic nervous control in chronic renal failure. Nephron.

[8] Kavelaars A, van de Pol M, Zijlstra J, Heijnen CJ (1997). Beta 2-adrenergic activation enhances interleukin-8 production by human monocytes. J Neuroimmunol.

[9] Speidl WS, Toller WG, Kaun C, Weiss TW, Pfaffenberger S, Kastl SP (2004). Catecholamines potentiate LPS-induced expression of MMP-1 and MMP-9 in human monocytes and in the human monocytic cell line U937: possible implications for peri-operative plaque instability. FASEB J.

[10] Borovikova LV, Ivanova S, Zhang M, Yang H, Botchkina GI, Watkins LR (2000). Vagus nerve stimulation attenuates the systemic inflammatory response to endotoxin. Nature.

[11] Wang H, Yu M, Ochani M, Amella CA, Tanovic M, Susarla S (2003). Nicotinic acetylcholine receptor alpha7 subunit is an essential regulator of inflammation. Nature.

[12] Vonend O, Rump LC, Ritz E (2008). Sympathetic overactivity--the Cinderella of cardiovascular risk factors in dialysis patients. Semin Dial.

[13] Ferchland A, Rettkowski O, Pönicke K, Deuber HJ, Osten B, Brodde OE (1998). Effects of uremic plasma on alpha- and beta-adrenoceptor subtypes. Nephron.

[14] Malik S, Winney RJ, Ewing DJ (1986). Chronic renal failure and cardiovascular autonomic function. Nephron.

[15] Vita G, Bellinghieri G, Trusso A, Costantino G, Santoro D, Monteleone F (1999). Uremic autonomic neuropathy studied by spectral analysis of heart rate. Kidney Int.

[16] Giordano M, Manzella D, Paolisso G, Caliendo A, Varricchio M, Giordano C (2001). Differences in heart rate variability parameters during the post-dialytic period in type II diabetic and non-diabetic ESRD patients. Nephrol Dial Transplant.

[17] Tong Y-Q, Hou H-M (2007). Alteration of heart rate variability parameters in nondiabetic hemodialysis patients. Am J Nephrol.

[18] Drawz PE, Babineau DC, Brecklin C, He J, Kallem RR, Soliman EZ (2013). Heart Rate Variability Is a Predictor of Mortality in Chronic Kidney Disease: A Report from the CRIC Study. Am J Nephrol.

[19] Chandra P, Sands RL, Gillespie BW, Levin NW, Kotanko P, Kiser M (2012). Predictors of heart rate variability and its prognostic significance in chronic kidney disease. Nephrol Dial Transplant.

[20] Chan CT, Chertow GM, Daugirdas JT, Greene TH, Kotanko P, Larive B (2014). Effects of daily hemodialysis on heart rate variability: results from the Frequent Hemodialysis Network (FHN) Daily Trial. Nephrol Dial Transplant.

[21] Chan CT, Levin NW, Chertow GM, Larive B, Schulman G, Kotanko P (2010). Determinants of cardiac autonomic dysfunction in ESRD. Clin J Am Soc Nephrol.

[22] Heart rate variability. Standards of measurement, physiological interpretation, and clinical use. Task Force of the European Society of Cardiology and the North American Society of Pacing and Electrophysiology. Eur Heart J. 1996;17(3):354–81. 8737210

[23] Goldstein DS, Bentho O, Park M-Y, Sharabi Y (2011). Low-frequency power of heart rate variability is not a measure of cardiac sympathetic tone but may be a measure of modulation of cardiac autonomic outflows by baroreflexes. Exp Physiol.

[24] Low PA, Benrud-Larson LM, Sletten DM, Opfer-Gehrking TL, Weigand SD, O'Brien PC (2004). Autonomic symptoms and diabetic neuropathy: a population-based study. Diabetes Care.

[25] Valensi P, Pariès J, Attali JR (2003). Cardiac autonomic neuropathy in diabetic patients: influence of diabetes duration, obesity, and microangiopathic complications—the french multicenter study. Metabolism.

[26] Brück K, Stel VS, Gambaro G, Hallan S, Völzke H, Ärnlöv J (2015). CKD Prevalence Varies across the European General Population. J Am Soc Nephrol.

[27] Whiting PJ, Vincent A, Schluep M, Newsom-Davis J (1986). Monoclonal antibodies that distinguish between normal and denervated human acetylcholine receptor. J Neuroimmunol.

[28] Roberts A, Lang B, Vincent A, Newsom-Davis J (1992). Search for cross-reactive idiotypes on monoclonal and myasthenia gravis acetylcholine receptor antibodies. Autoimmunity.

[29] Heidenreich F, Vincent A, Newsom-Davis J (1988). Differences in fine specificity of anti-acetylcholine receptor antibodies between subgroups of spontaneous myasthenia gravis of recent onset, and of penicillamine induced myasthenia. Autoimmunity.

[30] Dekker JM, Schouten EG, Klootwijk P, Pool J, Swenne CA, Kromhout D (1997). Heart rate variability from short electrocardiographic recordings predicts mortality from all causes in middle-aged and elderly men. The Zutphen Study. Am J Epidemiol.

[31] de Bruyne MC, Kors JA, Hoes AW, Klootwijk P, Dekker JM, Hofman A (1999). Both decreased and increased heart rate variability on the standard 10-second electrocardiogram predict cardiac mortality in the elderly: the Rotterdam Study. Am J Epidemiol.

[32] van Bemmel JH, Kors JA, van Herpen G (1990). Methodology of the modular ECG analysis system MEANS. Methods Inf Med.

[33] Kurata C, Uehara A, Sugi T, Ishikawa A, Fujita K, Yonemura K (2000). Cardiac autonomic neuropathy in patients with chronic renal failure on hemodialysis. Nephron.

[34] Faul F, Erdfelder E, Lang A-G, Buchner A (2007). G*Power 3: a flexible statistical power analysis program for the social, behavioral, and biomedical sciences. Behav Res Methods.

[35] Faul F, Erdfelder E, Buchner A, Lang A-G (2009). Statistical power analyses using G*Power 3.1: tests for correlation and regression analyses. Behav Res Methods.

[36] Stein PK, Domitrovich PP, Hui N, Rautaharju P, Gottdiener J (2005). Sometimes higher heart rate variability is not better heart rate variability: results of graphical and nonlinear analyses. J Cardiovasc.

[37] Reyes del Paso GA, Langewitz W, Mulder LJM, van Roon A, Duschek S (2013). The utility of low frequency heart rate variability as an index of sympathetic cardiac tone: a review with emphasis on a reanalysis of previous studies. Psychophysiology.

[38] Bootsma M, Swenne CA, Janssen MJA, Manger Cats V, Schalij MJ (2003). Heart rate variability and sympathovagal balance: pharmacological validation. Neth Heart J.

[39] Kuss O, Schumann B, Kluttig A, Greiser KH, Haerting J (2008). Time domain parameters can be estimated with less statistical error than frequency domain parameters in the analysis of heart rate variability. J Electrocardiol.

[40] Fukuta H, Hayano J, Ishihara S, Sakata S, Mukai S, Ohte N (2003). Prognostic value of heart rate variability in patients with end-stage renal disease on chronic haemodialysis. Nephrol Dial Transplant.

[41] Mylonopoulou M, Tentolouris N, Antonopoulos S, Mikros S, Katsaros K, Melidonis A (2010). Heart rate variability in advanced chronic kidney disease with or without diabetes: midterm effects of the initiation of chronic haemodialysis therapy. Nephrol Dial Transplant.

[42] Racek J, Králová H, Trefil L, Rajdl D, Eiselt J (2006). Brain natriuretic peptide and N-terminal proBNP in chronic haemodialysis patients. Nephron Clin Pract.

[43] Selim G, Stojceva-Taneva O, Spasovski G, Georgievska-Ismail L, Zafirovska-Ivanovska B, Gelev S (2011). Brain natriuretic peptide between traditional and nontraditional risk factors in hemodialysis patients: analysis of cardiovascular mortality in a two-year follow-up. Nephron Clin Pract.

[44] Psychari SN, Sinos L, Iatrou C, Liakos G, Apostolou TS (2005). Relations of inflammatory markers to lipid levels and autonomic tone in patients with moderate and severe chronic kidney disease and in patients under maintenance hemodialysis. Clin Nephrol.

[45] Kuroki K, Takahashi HK, Iwagaki H, Murakami T, Kuinose M, Hamanaka S (2004). beta2-adrenergic receptor stimulation-induced immunosuppressive effects possibly through down-regulation of co-stimulatory molecules, ICAM-1, CD40 and CD14 on monocytes. J Int Med Res.

[46] Girndt M, Sester U, Kaul H, Köhler H (1998). Production of proinflammatory and regulatory monokines in hemodialysis patients shown at a single-cell level. J Am Soc Nephrol.

[47] Sester U, Sester M, Heine G, Kaul H, Girndt M, Köhler H (2001). Strong depletion of CD14(+)CD16(+) monocytes during haemodialysis treatment. Nephrol Dial Transplant.

[48] Ulrich C, Heine GH, Garcia P, Reichart B, Georg T, Krause M (2006). Increased expression of monocytic angiotensin-converting enzyme in dialysis patients with cardiovascular disease. Nephrol Dial Transplant.

[49] Ulrich C, Heine GH, Seibert E, Fliser D, Girndt M (2010). Circulating monocyte subpopulations with high expression of angiotensin-converting enzyme predict mortality in patients with end-stage renal disease. Nephrol Dial Transplant.

[50] Ulrich C, Seibert E, Heine GH, Fliser D, Girndt M (2011). Monocyte angiotensin converting enzyme expression may be associated with atherosclerosis rather than arteriosclerosis in hemodialysis patients. Clin J Am Soc Nephrol.

[51] Oduah EI, Linhardt RJ, Sharfstein ST. Heparin: Past, Present, and Future. Pharmaceuticals (Basel). 2016;9(3):38. doi:10.3390/ph9030038. 10.3390/ph9030038 PMC5039491 27384570

[52] Young E (2008). The anti-inflammatory effects of heparin and related compounds. Thromb Res.

