# Supplementary material for: Association between autonomic nervous dysfunction and cellular inflammation in end-stage renal disease
Source: BMC Cardiovasc Disord. 2016 Nov 3;16:210. doi: 10.1186/s12872-016-0385-1 (PMC5094069; doi:10.1186/s12872-016-0385-1)
Supplement: Additional file 1: Table S1. — Gender- and age-adjusted means ± SE of ECG analysis parameters in healthy controls and dialysis patients. (DOCX 13 kb) [file 12872_2016_385_MOESM1_ESM.docx]

**Additional file 1: Table S1.** Gender- and age-adjusted means ± SE of ECG analysis parameters in healthy controls and dialysis patients.

|  | **Controls**  (N=15) | **Diabetic HD**  (N=14) | **Nondiabetic HD**  (N=14) |
| --- | --- | --- | --- |
| **Heart rate**  (/min) | 65 ± 4 | 80 ± 4 | 79 ± 3* |
| **SDNN**  (ms) | 44.2 ± 6.4 | 19.2 ± 6.7 | 18 ± 5.6* |
| **pNN50**  (%) | 7.9 ± 3.0 | 4.3 ± 3.2 | 1.5 ± 2.7 |
| **VLF**  (s^2^ x10^-3^) | 0.9 ± 0.2 | 0.3 ± 0.2* | 0.3 ± 0.2* |
| **LF**  (s^2^ x10^-3^) | 1.0 ± 0.4 | 0.3 ± 0.4 | 0.1 ± 0.3 |
| **HF**  (s^2^ x10^-3^) | 0.7 ± 0.2 | 0.3 ± 0.2 | 0.1 ± 0.2 |
| **LF/HF** | 1.5 ± 0.6 | 1.3 ±0.6 | 2.1 ± 0.5 |

*=p<0.05 vs. controls; SE: Standard error.
